# Piezo in the eye: expression, distribution and roles in ocular diseases

**DOI:** 10.3389/fphys.2025.1651258

**Published:** 2025-08-12

**Authors:** Li Zhu, Shouyan Ren, Shuang He, Meifang Liu, Fei Hou

**Affiliations:** ^1^ Department of Pharmacy, The Affiliated Hospital of Qingdao University, Qingdao, Shandong, China; ^2^ Department of Otolaryngology, The Affiliated Hospital of Qingdao University, Qingdao, Shandong, China; ^3^ School of Medicine and Pharmacy, Ocean University of China, Qingdao, Shandong, China; ^4^ School of Pharmacy, Jining Medical University, Rizhao, Shandong, China

**Keywords:** Piezo channels, glaucoma, IOP, RGC, ONH

## Abstract

Piezo channels are currently known to be the most sensitive molecular mechanoreceptors. Piezo can respond to membrane tension, sag, shear force, tensile and other mechanical stimuli, produces fast inactivation, small conductance, and low threshold current. In eukaryotic cells, Piezo has two family members: Piezo1 and Piezo2. Functionally, Piezo1 detects whole-membrane tension changes, including swelling and compression. Piezo2 is more likely to sense specific mechanical stimuli, including touch and airway stretching. In the ocular system, Piezo1 and Piezo2 are expressed across various cells and tissues. This article provides a comprehensive review of the expression, distribution, and function of Piezo channels in ocular tissues, offering novel insights for the treatment of eye diseases.

## 1 Introduction

Cells and organisms rely on force sensing to interact with their environment (Inman and Smutny, 2021). The eye is constantly subjected to mechanical stress induced by normal eye activities, including blinking, eye pulse and eye rubbing and so on. These activities can lead to compression of the eyeball and drastic fluctuations in intraocular pressure (IOP) (Hirt and Liton, 2017; Killer and Pircher, 2018). While the exact mechanisms of how the eye adapts to these changes are unclear, mechanosensitive ion channels may play important roles in ocular function and intraocular pressure regulation. Several studies reported the expression distribution and roles of Piezo channels in different ocular tissues.

Piezo channels, identified by Ardem Patapoutian’s team in 2010, are mechanically sensitive ion channels comprising Piezo1 and Piezo2 (Coste et al., 2010). The channel has attracted extensive attention and research enthusiasm worldwide, and Patapoutian was awarded the 2021 Nobel Prize in Physiology for his discovery of Piezo2 as a tactile receptor. Piezo channels play a very important role in mechanical force transduction and are more sensitive to mechanical stimulation than TRPV4 (Transient Receptor Potential Vanilloid 4), TREK1 (Tandem of P domains in a Weak Inward rectifying K^+^ channel) and other mechanosensitive ion channels (Rocio Servin-Vences et al., 2017). Previous studies have demonstrated that Piezo1 functions as a hemodynamic sensor essential for vascular development and blood pressure regulation (Li et al., 2014). Moreover, Piezo1 also has important roles in maintaining homeostasis of epithelial cell numbers (Eisenhoffer et al., 2012; Gudipaty et al., 2017), the red blood cells volume regulation, cell migration and differentiation (He et al., 2018; Ellefsen et al., 2019),neuronal axon growth (Song et al., 2019), neural stem cell fate (Li et al., 2022), mechano-transduction in cartilage (Rocio Servin-Vences et al., 2017), pressure induced pancreatitis (Romac et al., 2018), and control of urinary osmolarity (Martins et al., 2016). Piezo2 acts as a key mechano-transducer in response to gentle touch sensation (Chesler et al., 2016), proprioception (Woo et al., 2015), secretion of 5-HT (Wang et al., 2017), and airway stretch and lung inflation-induced apnoea (Nonomura et al., 2017). The genetic mutation of *piezo* gene leads to a variety of human genetic diseases, such as red cell shriveled syndrome (Faucherre et al., 2014), lymphedema (Fotiou et al., 2015; Lukacs et al., 2015), and distal joint contracture (Coste et al., 2013; McMillin et al., 2014; Okubo et al., 2015).

Yidan Chen et al. reviewed the role of Piezo1 ion channel in the pathogenesis of glaucoma (Chen et al., 2022). Given Piezo’s broad expression in ocular tissues, it may contribute to other ophthalmic pathologies. In this paper, we focused on the expression distribution of Piezo in the eye and its potential pathophysiological function by reviewed the relevant literature published in recent years, which provided a new perspective for the diagnosis and treatment of ocular diseases.

## 2 Features of piezo channels

Piezo, an ion channel known for its ability to sense and transduce mechanical stimulation, exhibits several distinctive features when compared to other families of mechanosensitive ion channels. The most notable feature of Piezo is its structural uniqueness, which is characterized by ahomotrimeric structure, distinguishing it from all known ion channels and other protein classes (Nosyreva et al., 2021). Xiao Bailong et al. presented a detailed analysis of the high-resolution cryo-electron microscopy structure of the full-length (2,547 amino acids) mouse Piezo1, proposing a trimeric propeller-like structure model, with the extracellular domains resembling three distal blades and a central cap.

Another notable characteristic is the high degree of conservation in the *Piezo* gene sequence across various species. Mechanosensitive ion channels such as NompC, a member of the TRP family, respond to mechanical stimuli in organisms like *Drosophila melanogaster* and nematodes; however, their orthologous genes in mammals do not exhibit mechanosensitivity (Kim et al., 2012). In contrast, the *Piezo* gene in *Drosophila* remains responsive to mechanical stimuli and can generate mechanically-activated (MA) current akin to those of human *Piezo* channels when expressed in human cells (Coste et al., 2012). Furthermore, Piezo channels are widely expressed across diverse species, spanning animals, plants, and protozoa. Within the same species, Piezo1 and Piezo2 exhibit broad tissue and organ distribution, highlighting their functional versatility (Wu et al., 2017).

Functionally, the critical role of Piezo channels is underscored by the fact that the absence of Piezo1 in mice results in embryonic lethality around day 10 due to impaired blood vessel formation (Ranade et al., 2014). Similarly, the absence of Piezo2 leads to neonatal death within 24 h due to the failure to establish a respiratory reflex (Nonomura et al., 2017). Piezo2 is recognized as a mechanosensitive ion channel essential for light touch perception. Conditional knockout of Piezo2 in the skin abolishes light touch responses in mice.

## 3 Expression and physiological functions of piezo in the eye

### 3.1 Cornea

The cornea is a transparent, avascular tissue at the anterior ocular surface that is densely innervated by sensory nerve endings (Marfurt et al., 2010). Early studies have identified three main types of sensory neurons in the corneal epithelium: mechano-nociceptors, polymodal nociceptors, and cold-sensing neurons (González-González et al., 2017). These neurons play a crucial role in quickly detecting external mechanical stimuli to protect the corneal epithelium from damage. In 2014, utilizing retrograde tracing, immunohistochemistry, and *in situ* hybridization assays, Bron et al. characterized the expression of Piezo2 channels in sensory neurons (Bron et al., 2014). Their findings revealed that Piezo2 expression is localized to a subset of mechano-nociceptors within corneal afferent neurons, distinct from polymodal nociceptors or cold-sensing neurons. The researchers proposed that Piezo2 may serve as a promising candidate for transducing noxious mechanical stimuli (Bron et al., 2014). Jorge Fernandez-Trillo, Ana Gomis, and their colleagues identified Piezo2 expression in both pure mechanosensory and polymodal nociceptor corneal neurons, and successfully recorded mechanically-activated (MA) currents. Their experiments with Piezo2-CKO (conditional knock out) mice strongly indicate that Piezo2 plays a significant role in the early activation of corneal nociceptor neurons (Fernández-Trillo et al., 2020). These results offer a theoretical foundation for the potential topical targeting of Piezo2 as a treatment for ocular surface pain.

### 3.2 Ciliary body

Aqueous humor (AH) is a clear liquid in the eye that plays an important role in maintenance of intraocular pressure and provide nutrition to intraocular structures including, the cornea and lens (Murthy et al., 2015). It is secreted by the ciliary body, which contains two layers: Non-pigmented epithelial cells (NPE) and Pigmented epithelial cells (PE) (Skalicky, 2016). Currently, the commonly used medicine of Glaucoma, such as Timolol and Afagon, can reduced intraocular pressure by decreasing the production of aqueous humor (Liu et al., 2022).

Several studies have reported that the expression of Piezo1 in ciliary body. Ying Zhu et al. observed robust expression of *piezo1*mRNA and protein but only rare *piezo2* mRNA in the ciliary body epithelium using single-molecule fluorescence *in situ* hybridization (smFISH) and immunohistochemical (IHC) analysis of PIEZO1^tdTomato^ and PIEZO2^GFP^ reporter mice (Zhu et al., 2023). We also found that both PIEZO1 and PIEZO2 were expressed in non-pigmentary epithelium of the ciliary body (Zhu et al., 2021). In addition, Jingwang Fang et al. reported that Piezo1 and Piezo2 were highly expressed in ciliary muscle (Fang et al., 2021). Identification of the role of Piezo in aqueous humor production and intraocular pressure (IOP) modulation is warranted in future work, as this mechanosensitive channel may represent a novel intervene target for AH production.

### 3.3 The trabecular meshwork

The trabecular network (TM) is a specialized tissue resided in the iris corneal angle which is the main site of aqueous humor outflow (Buffault et al., 2020). The trabecular network itself is mechanically sensitive, and the natural mechanosensitive molecules in trabecular network maybe play an important role in sensing and recognizing changes of IOP (Jing et al., 2024).

As early as 2014, Vu T. Tran, et al. reported that human trabecular network tissues and cells expressed 11 types of mechanosensitive molecules, including PIEZO1 and PIEZO2 (Tran et al., 2014). David et al. detected Piezo1 expression in mouse (mTM) and human (hTM) trabecular meshwork cells using RT-PCR, immunohistochemistry, and Piezo1P1-tdT reporter mice, in which a tdTomato fluorescent reporter was inserted upstream of the Piezo1 stop codon in exon 51 (Yarishkin et al., 2021). We also detected the expression of both PIEZO1 and PIEZO2 in hTM. While PIEZO1 exhibited similar expression levels across different TM layers, PIEZO2 was predominantly localized in the uveal TM layer. Above all, we identified the functional expression of PIEZO1 in the trabecular cells used electrophysiological experiments. Specially, whole-cell patch-clamp recordings showed that Yoda1 (the selective Piezo1 agonist) can increase the peak value of a fast activation current induced by mechanical stimulation of TM cells and can slow down their inactivation rate. The Piezo1-induced current was suppressed by L-GsMTx4 treatment or abolished by Piezo1 shRNA knockdown (Zhu et al., 2021).

However, it remains unclear whether Piezo can respond mechanical forces in the TM, and whether it has regulatory effects on AH outflow and IOP. There is a positive feedback regulatory mechanism between Piezo1 (a molecular receptor for force) and force in osteoblasts, that is to say, the expression of Piezo1 increased when the cultured osteoblasts were mechanically stretched. We found that the expression of PIEZO1 also increased significantly when cyclic mechanical stretching was applied to cultured human trabecular cells (Wang et al., 2020). In addition, Takatoshi Uchida et al. demonstrated that in human trabecular meshwork cells, both mechanical stretch and treatment with the PIEZO1 agonist Yoda1 induced a PIEZO1-dependent calcium influx, accompanied by the subsequent release of arachidonic acid and prostaglandin E2 (PGE2) (Uchida et al., 2021).

To assess the direct effect of Piezo activation on regulating conventional outflow, we measured the aqueous outflow facility by delivered L-GsMTx4, an inhibitor of mechanically sensitive ion channels (MSCs), into the anterior chamber of mouse eyes. We found that infusion of 3.3 μM L-GsMTx4 into the isolated eyeball had little effect on the outflow facility of aqueous humor, but infusion of 10 μM L-GsMTx4 caused a significant decrease in the outflow facility of aqueous humor (Zhu et al., 2021). Consistent with our findings, Oleg Yarishkin et al. also discovered that TM-dependent fluid drainage from the anterior eye showed significant inhibition by L-GsMTx4 (Yarishkin et al., 2021).

Several studies have reported that Piezo1 may potentially have arole in regulating IOP. Wataru Morozumi et al. reported that Piezo1 activation inhibited hTM cells proliferation, and reduced expression level of fibronectin (Morozumi et al., 2021). What is more, the eye drop containing Yoda1 reduced the IOP in mice, and also decreased fibronectin expression level around the TM. We also determined the role and mechanism of Piezo channel in the regulation of IOP. Confusingly, there was no significant change in mouse IOP after anterior chamber injection of L-GsMTx4 inhibiting the function of mechanosensitive channels. In addition, we explored the role of Piezo in the eye’s rapid response to IOP changes using ocular compliance model. We found that L-GsMTx4 at 10 μM increased the eye compliance, but Yoda1 at 20 μM had no significant effect on the eye compliance (Zhu et al., 2021). Given previous studies of Piezo channel and IOP regulation had only focused on either Piezo1 or Piezo2, in future studies, we plan to focus on explore how different mechnosensitive ion channels interact to modulate IOP.

### 3.4 Retina

The retina, comprising the sensory nerve layer and the pigment epithelium, serves as the primary light-detecting tissue in mammals and plays a crucial role in vision (Ptito et al., 2021). Degeneration and death of retinal ganglion cells (RGCs) can result in visual impairment or blindness (Liu and Margeta, 2019). Previous studies have demonstrated that RGCs express transient receptor potential channels of the vanilloid subtype (TRPV1 and TRPV4), both of which are implicated in RGCs apoptosis (Sappington et al., 2009; Ryskamp et al., 2011; Gao et al., 2019). However, there are limited reports regarding the Piezo channels in the retina. Wataru Morozumi et al. identified the expression sites of Piezo channels in the murine retina. They discovered that Piezo2 is extensively expressed throughout the retina, while Piezo1 is localized specifically to the ganglion cell layer (GCL) (Morozumi et al., 2020). Furthermore, the expression level of Piezo2 was found to be elevated in a retinal disorder mouse model induced by high intraocular pressure (IOP). Unlike Wataru Morozumi’s findings, Ying Zhu et al. reported that *piezo2* mRNA was not detected in the inner nuclear layer (INL) or the outer nuclear layer (ONL), with only a few cells expressing *piezo2* mRNA in the GCL. Furthermore, they observed *piezo1* mRNA in the GCL and INL, with a low level of *piezo1* mRNA in the ONL (Zhu et al., 2023). We exclusively examined Piezo2 expression in the retina and observed its distribution throughout various retinal layers.

Manuela Völkner and colleagues developed a human retinal organoid system that replicates various parameters of the human retina, including aspects of the macula, to model a complex interplay of photoreceptor and glial pathologies (Völkner et al., 2022). Their research demonstrated that the combined application of tumor necrosis factor (TNF) and heparin-binding EGF-like growth factor (HBEGF) is sufficient to induce photoreceptor degeneration. Furthermore, they proposed that photoreceptor neuro degeneration occurs via cell extrusion, noting the intriguing role of the Piezo1 activator Yoda1 in inducing photoreceptor extrusion. Future research is needed to determine whether Piezo channels influence neuronal physiology or visual processing.

### 3.5 Optic nerve head

The axons of ganglion cells form the layer of nerve fibers that converge to create the optic nerve, serving as the foundation for various visual functions (De Moraes, 2013). The potential direct sensitivity of optic nerve head (ONH) astrocytes to pressure or stretch has been investigated using cell culture systems. Several candidate molecules have been proposed to impart mechanosensitivity to optic nerve astrocytes (Sigal et al., 2005). Notably, studies have reported the expression of Piezo1 and Piezo2 in the astrocytes of the mouse ONH, with elevated levels of Piezo2 observed in rodent glaucoma model, specifically DBA/2J. Similarly, research by Choi et al. identified the expression of Piezo1 in the mouse ONH, suggesting its role in astrocytes’ response to traumatic or glaucomatous injury (Choi et al., 2015). Koser et al. demonstrated that the growth of retinal ganglion cell (RGC) axons in *Xenopus* was disrupted when Piezo1 expression was reduced (Koser et al., 2016). Furthermore, Yue Wan et al. showed that Piezo1 is necessary but insufficient for ONH astrocyte proliferation, as revealed by gain- and loss-of-function experiments *in vitro*. Genetic ablation of Piezo1 induced cell cycle arrest through a mechanotransduction pathway involving impaired nuclear translocation of YAP and consequent downregulation of cell cycle regulators, including cyclin D1 and c-Myc (Wan et al., 2023). The establishment of this novel ‘retinal mechanosensation’ research direction holds significant implications for elucidating the pathogenesis of various retinal diseases and developing innovative therapeutic strategies.

### 3.6 Lens

The lens plays a critical role in focusing light onto the retina, making its transparency essential for the formation of normal vision (Liu et al., 2017). Any factor compromising lens transparency can result in visual impairment. It is hypothesized that mechanosensitive ion channel activities influence changes in lens shape and clarity (Nakazawa et al., 2021). In a study by Wataru Morozumi et al., the expression of Piezo1 and Piezo2 in mouse lens epithelial cells was identified using immunostaining techniques (Morozumi et al., 2020). However, the role of Piezo channels in lens function remains poorly understood.

## 4 Piezo and related ocular disease

### 4.1 Dry eye disease (DED)

Dry eye disease (DED) is a multifactorial condition marked by ocular discomfort and irritation. Despite advancements have been made in understanding this chronic ocular surface disease, its pathological mechanisms remain incompletely elucidated (Galor et al., 2018; Shimazaki, 2018). Initially regarded as primarily a tear production abnormality, DED is now recognized to have significant pathophysiological links with autoimmune disorders (Marsovszky et al., 2013). Multiple studies have suggested that Piezo2 channelopathy may contribute to autoimmune disease pathogenesis (Sonkodi, 2022; Sonkodi et al., 2022b). Sonkodi et al. demonstrated a potential neuroimmunological link between DED and rheumatoid arthritis through the chronic Piezo2 channelopathy-induced two-pore-domain potassium (K2P-TASK1) signaling axis (Sonkodi et al., 2023).

Recent research has indicated that DED encompasses a spectrum that includes neuropathic corneal pain. Nevertheless, DED is predominantly characterized by superficial disturbances and is frequently devoid of pain (Dieckmann et al., 2022). Sonkodi and Hortobágyi et al. proposed a quad-phasicnon-contact injury model (a biomechanical simulation system for studying trauma mechanisms in neural, ocular, and other biological tissues) of the Piezo2 receptor to elucidate this paradox (Sonkodi et al., 2022a). Epidemiological data indicate a 2-3 times higher incidence of DED in women compared to men. Sonkodi et al. attribute this disparity to women’s heightened Piezo2-mediated mechanosensitivity in corneal sensory receptors. They also posited that the NGF (never growth factor)-TrkA (tyrosine kinase-A) axis contributes to the microinjury of the Piezo2 receptor, suggesting that the interaction between NGF-TrkA and Piezo2 may account for the observed sex differences in DED. Investigating the precise role of Piezo channels in lacrimal gland physiology is crucial for elucidating the mechanobiological mechanisms underlying DED pathogenesis.

### 4.2 Glaucoma

Glaucoma is a progressive optic neuropathy characterized by specific clinical manifestations, including elevated intraocular pressure (IOP), visual field defects, and optic nerve damage. Based on the morphology of the anterior chamber angle, glaucoma is classified into two primary categories: Primary Open-Angle glaucoma (POAG) and Primary Angle-Closure glaucoma (PACG) (Weinreb et al., 2014). Intraocular pressure refers to the fluid pressure within the eye, which is maintained by the dynamic equilibrium of aqueous humor (AH) circulation. An overproduction of AH or increased resistance to its outflow results in elevated IOP, a major risk factor for glaucoma (Braunger et al., 2015). Clinical studies indicate that in most glaucoma patients, AH production is normal, and the primary cause of increased IOP is the obstruction of AH outflow. Given that 80% of AH is discharged through the trabecular meshwork, abnormalities in its structure and function are significant contributors to elevated IOP (van Zyl et al., 2020).

Mutations in the PIEZO gene are implicated in a range of human genetic disorders, including red cell shriveled syndrome, lymphedema, and distal joint contracture. Given that PIEZO channels have been found in the trabecular meshwork of human eyes, coupled with glaucoma is clearly a pressure-sensitive disease, further investigations of *piezo* variants in glaucoma are still warranted. Sally L. Baxter and colleagues conducted an analysis of whole genome sequences from 1,565 participants of African descent (Baxter et al., 2020). They identified the PIEZO1 E756del gain-of-function variant as the most prevalent polymorphism in this population, although no statistically significant differences were observed in glaucoma phenotypes. Wendy W. Liu et al. explored the associations between mechanosensitive ion channel gene variants and primary open-angle glaucoma (POAG) using data from independent human genetic datasets (Liu et al., 2023). They discovered two rare PIEZO1 coding variants with protective effects in the NEIGHBOR dataset.

In conclusion, substantial research is still required to advance our understanding of the role of PIEZO and mechano-transduction in the pathophysiology and management of glaucoma and other related conditions.

### 4.3 Myopia

The global prevalence of myopia is rising at an alarming rate; however, the etiology and pathogenesis of myopia have not been elucidated (Baird et al., 2020). Weiqi Zhong and colleagues explored the relationship between the Piezo1 channel and myopia. They found that the Piezo1 channel was activated in the retinas of guinea pigs with form deprivation myopia (FDM) and may play a role in the development of myopia by modulating intraocular reactive oxygen species (ROS) levels (Zhong et al., 2023).

### 4.4 Cataract and presbyopia

Cataract and presbyopia are two of very common visual defects that are caused by the loss of clarity and focusing abilities, respectively (Fricke et al., 2018). Ariana Allen et al. investigated the effects of lens shape change and the activity of Piezo channels on mouse lens myosin II activity (Allen et al., 2022). *Ex vivo* experiments (Freshly isolated mouse lenses are used in *ex vivo* experiments) demonstrated that changes in lens shape, induced by external mechanical loading, resulted in a reduction of calcium-dependent myosin II activity. Activation of Piezo1 via Yoda1 for 1 hour increased the levels of phosphorylated myosin light chain (MLC); however, prolonged Yoda1 treatment (6 and 24 h) led to lens opacification, which was associated with elevated calpain activity and degradation of membrane proteins in *ex vivo* mouse lenses. Conversely, inhibition of Piezo1 withL-GsMTx4 decreased MLC phosphorylation but did not alter the lens tensile characteristics. Research on Piezo channels in cataract pathogenesis remains relatively limited, making targeted ‘mechano-protective therapy’ a potential frontier in this field.

## 5 Summary and perspectives

**FIGURE 1:**
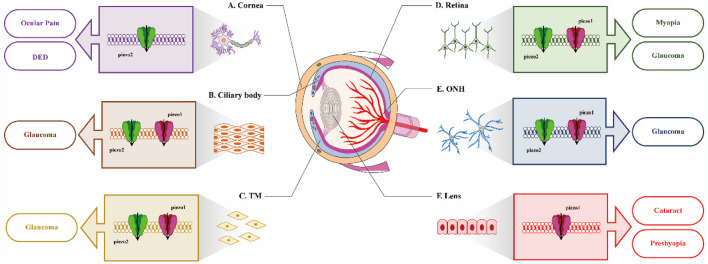
Distribution of Piezo in different ocular tissues and itspotential role in diseases. **(A)** In cornea, inhibition of Piezo2 may be beneficial for the treatment of ocular pain and DED; **(B)** and **(C)**. In the ciliary body and trabecular meshwork (TM), the expression of both Piezo1 and Piezo2 suggests their potential involvement in IOP regulation; **(D)** and **(E)**. In retina and ONH, identifying the function of Piezo channels on visual processing would be very interesting; **(F)** In lens, the activation of Piezo1 by Yoda1 leads to opacification of mouse lens.

In conclusion, the extensive expression of Piezo channels across various ocular tissues indicates their potential significance in both physiological and pathological processes, warranting further investigation. We attempt to summarize the expression patterns and potential functions of Piezo in the eye through Figure 1. Elucidating the role of Piezo channels in the pathogenesis of ocular diseases may offer novel insights into potential therapeutic strategies. However, current investigations in this field face two major methodological constraints: the inherent limitations of singular detection approaches and the current unavailability of Piezo-specific pharmacological modulators (both agonists and antagonists). Consequently, numerous aspects require additional exploration in future studies.

1. Investigating the alterations in Piezo expression among glaucoma patients is essential for establishing a clinical foundation for targeting Piezo as a therapeutic intervention for glaucoma.
2. Developing high intraocular pressure (IOP) mouse models with Piezo1 or Piezo2 conditional knockouts to examine the effects of Piezo on IOP under conditions of elevated IOP.
3. Identifying specific inhibitors and agonists of Piezo1 and Piezo2 is crucial to advance the understanding of the functions and biophysical properties of the Piezo channels.
4. Examining the interactions among various mechanosensitive ion channels in modulating IOP is of significant importance for the prevention and treatment of glaucoma.
5. Investigating the role of Piezo2 in corneal sensory neurons in response to changes in the external environment presents an intriguing area of study.

Functioning as biological ‘mechanosensors’ in the eye, Piezo channels contribute not only to understanding disease mechanisms but more importantly to advancing precision medicine development.

## Abbreviations

IOP, Intraocular pressure; TRV4, Transient receptor potential vanilloid 4; TREK1, Two Pore-Domain Potassium Channels 1; 5-HT, 5-hydroxytryptamine; MA, Mechanically-activated; CKO, conditional knock out; AH, Aqueous humor; PE, Pigmented Epithelial; NPE, Non-pigmented epithelial sone; smFISH, single-molecule fluorescence *in situ* hybridization; TM, trabecular network; PGE2, Prostaglandin E2; MSCs, mechanically sensitive ion channels; RGC, Retinal Ganglion Cell; GCL, ganglion cell layer; INL, Inner nuclear layer; ONL, Outer nuclear layer; TNF, tumor necrosis factor; HBEGF, heparin-binding EGF-like growth factor; YAP, yes-associated protein; DED, dry eye disease; K2P, two-pore-domain potassium; NGF, never growth factor; TrkA, tyrosine kinase-A; POAG, Primary open-angle glaucoma; ACG, Primary angle-closure glaucoma; FDM, form deprivation myopia; ROS, reactive oxygen species; MLC, myosin light chain
